# Novel Mutation in the SLC19A2 Gene in an Iranian Family with Thiamine-Responsive Megaloblastic Anemia: A Series of Three Cases

**DOI:** 10.4274/Jcrpe.969

**Published:** 2013-09-18

**Authors:** Nosrat Ghaemi, Martha Ghahraman, Mohammad Reza Abbaszadegan, Alireza Baradaran-Heravi, Rahim Vakili

**Affiliations:** 1 Department of Pediatric Endocrinology, Imam Reza Hospital, Mashhad University of Medical Sciences (MUMS), Mashhad, Iran; 2 Human Genetics Division, Immunology Research Center, and Medical Genetic Research Center (MGRC), Mashhad University of Medical Sciences (MUMS), Mashhad, Iran; 3 Child and Family Research Institute, Department of Medical Genetics, University of British Columbia, Vancouver, British Columbia, Canada

**Keywords:** Megaloblastic anemia, diabetes mellitus, hearing loss, SLC19A2

## Abstract

Thiamine-responsive megaloblastic anemia (TRMA) is a clinical triad characterized by megaloblastic anemia, non-autoimmune diabetes mellitus, and sensory-neural hearing loss. Mutations in the thiamine transporter gene, solute carrier family 19, member 2 (SLC19A2), have been associated with TRMA. Three pediatric patients from a large consanguineous Iranian family with hyperglycemia, anemia, and hearing loss were clinically diagnosed with TRMA. In all three patients, TRMA was confirmed by direct sequencing of the SLC19A2 gene that revealed a novel missense homozygous mutation c.382 G>A (p.E128K). This mutation results in the substitution of glutamic acid to lysine at position 128 in exon 2 and was not detected in 200 control chromosomes. Thiamine therapy reversed the anemia and alleviated the hyperglycemia in all three patients. We recommend sequence analysis of the SLC19A2 gene in individuals with a clinical triad of diabetes mellitus, hearing loss, and anemia. The administration of thiamine ameliorates the megaloblastic anemia and the hyperglycemia in patients with TRMA.

**Conflict of interest:**None declared.

## INTRODUCTION

Thiamine-responsive megaloblastic anemia (TRMA) or Rogers syndrome is a rare autosomal recessive disorder characterized by early-onset diabetes mellitus, anemia, and sensorineural deafness. Other less common abnormalities such as congenital heart disease, degeneration of the retina and the optic nerve, and stroke-like episodes have also been reported with this syndrome (1). Mutations in the SLC19A2 gene, encoding the thiamine transporter protein thiamine transporter 1 (THTR1), have been associated with TRMA (2,3,4). Although lifelong thiamine treatment is required for improving the hematologic and endocrine pathologies, no response has been reported for the neurological symptoms including the hearing loss (5,6). This study presents three children with TRMA from a large consanguineous Iranian family who demonstrated a novel mutation within the SLC19A2 gene. 

## REPORTS OF THE CASES

**Case 1**

**Figure 1:**
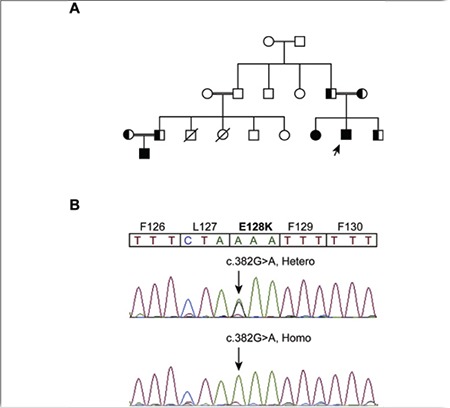
Family pedigree and SLC19A2 mutation analysis: A. The pedigree of the large consanguineous family with TRMA; B. The DNA sequencing chromatograms showing a G to A transition at codon sequence 382 in exon 2 of the SLC19A2 gene; Patients are homozygous for the mutation and the healthy members of the families are heterozygous

The propositus was the second child of healthy first-cousin parents. Hearing loss was noted since he was six months of age. The patient was referred to our hospital with growth restriction, megaloblastic anemia (Hb 10.1 g/dL, MCV 97.2) and hyperglycemia (FBS 191 mg/dL) at the age of 16 months. Audiometry revealed deep sensorineural hearing loss. Clinical diagnosis of TRMA was suspected and confirmed by DNA sequencing of the coding exons of the SLC19A2 gene. This revealed a novel homozygous mutation c.382G>A resulting in the substitution of glutamic acid to lysine at position 128 (p.E128K) of the SLC19A2 protein. Healthy members of the family including parents and a younger brother were heterozygous for this mutation (Figure 1). The c.382G>A missense mutation was absent in 200 unrelated control alleles confirmed by polymerase chain reaction followed by restriction fragment length polymorphism. Thiamine therapy ameliorated the patient’s anemia and hyperglycemia. In the patient’s most recent visit, his growth status, his hemoglobin and blood glucose levels were normal (Table 1). 

**Case 2**

The proposita was the elder sibling of case 1 (Figure 1). At age 3.9 years, she was referred to our hospital with failure to thrive, fever, and hyperglycemia. Laboratory findings revealed hyperglycemia (FBS 248 mg/dL) and megaloblastic anemia (Hb 11.5 g/dL, MCV 96). Audiometery showed sensorineural hearing loss. Analysis of her SLC19A2 gene was compatible with TRMA syndrome; thus, thiamine treatment was started. This ameliorated her anemia, but the hyperglycemia lingered and required insulin therapy (Table 1). 

**Case 3**

The propositus was the cousin of case 1. He was born at term by normal vaginal delivery as the only child of healthy second-cousin parents (Figure 1). The patient was found to be anemic at six months of age (Hb 8.7 g/dL, MCV 94) and at age one year developed hyperglycemia (FBS 170 mg/dL). Audiometry revealed sensorineural hearing loss (Table 1). Based on his positive family history, sequencing of the SLC19A2 gene was performed which revealed similar mutation to his affected cousins. Accordingly, oral treatment with thiamine was started, resulting in amelioration of his anemia and hyperglycemia (Table 1). In the patient’s most recent follow-up visit at age four years, his blood glucose and hemoglobin levels were normal.

## DISCUSSION

In this report, we described three patients with thiamine-responsive megaloblastic anemia. All 3 patients were the offspring of a large consanguineous Iranian family having a novel missense mutation in the SLC19A2 gene. TRMA, first described by Porter et al in 1969 (7), is associated with mutations in the SLC19A2 gene. This gene is a member of the solute carrier gene superfamily and encodes thiamine transporter 1 (8,9). THTR1 is a 497 amino acid protein with 12 transmembrane domains that serves as a saturable active thiamine transporter from the extracellular to intracellular space (10,11). In addition to the active transport mediated by THTR1, thiamine is also transported by another passive low-affinity and non-saturable mechanism which is intact in patients with TRMA syndrome (10). This explains the absence of vitamin B1 deficiency (beriberi) symptoms in these patients. It seems that a high concentration of intra-cellular thiamine is important for the function and integrity of certain tissues, such as islet cells of the pancreas, hematopoietic, and cochlear cells. Anemia is usually an early finding in TRMA syndrome. Unfortunately our patients had not been properly evaluated when they first presented. The classical hematologic profile in the TRMA syndrome is a macrocytic anemia which develops early in life and which is sometimes associated with thrombocytopenia or neutropenia. Sideroblastic anemia has also been reported (12). All of our patients presented with megaloblastic anemia and had no thrombocytopenia. Diabetes mellitus in TRMA syndrome differs from the typical type 1 diabetes. It is likely that the hyperglycemia in these patients is due to a thiamine deficiency in the pancreatic islet cells. The mutation in the high-affinity thiamine transporter gene, SLC19A2, supports this hypothesis (13).

The novel mutation observed in our patients, c.382G>A, leads to substitution of negatively charged R-group glutamic acid (pKa=4) by positively charged R-group lysine (pKa=10.5) at position 128 (E128K) in exon 2 of the SLC19A2 gene.

After the diagnosis of TRMA in our patients, treatment was initiated with a high dose of thiamine (200 mg/day) and continued with long-term thiamine supplementation (25-50 mg/day). Although significant improvement in the blood sugar profile occurred, low-dose insulin was still needed for diabetes control in patient 2, indicating that thiamine treatment may not fully correct the lack of insulin. The anemia disappeared after long-term treatment, but the hearing loss persisted in all three patients.

In summary, we presented three TRMA patients with a novel missense mutation in the SLC19A2 gene. Administration of thiamine in patients with TRMA ameliorates the megaloblastic anemia and diabetes mellitus. We recommend assessment of TRMA syndrome using SLC19A2 gene analysis in any diabetic patient with anemia or deafness. Also, we suggest prenatal diagnosis for high-risk pregnancies in families with TRMA-affected individuals. 

**Acknowledgement**

This study was supported by grants from Mashhad University of Medical Sciences. We gratefully acknowledge the contribution of the scientific collaborators of Human Genetics Department of Bu-Ali Research Institute of Mashhad University of Medical Sciences.

## Figures and Tables

**Table 1:**
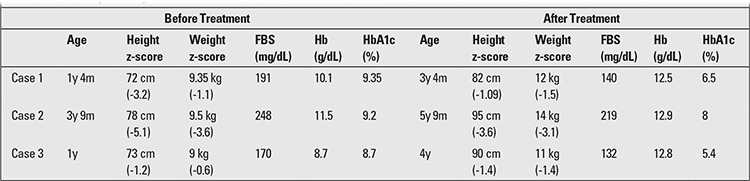
Laboratory data of patients before and after treatment with thiamine
